# Risk factors for thrombotic events in Philadelphia chromosome-negative myeloproliferative neoplasms: a retrospective analysis of 336 cases

**DOI:** 10.3389/fonc.2025.1639250

**Published:** 2025-09-02

**Authors:** Lingxiu Zhang, Xiufeng Wang, Man Yang, Youmei Zi, Yuan Zhang, Yan Guo, Guoqing Lv, Sun Wu, Yan Huang

**Affiliations:** ^1^ Department of Hematology, The First Affiliated Hospital of Xinxiang Medical University, Weihui, Henan, China; ^2^ Xinxiang Key Laboratory of Molecular Diagnosis and Treatment of Lymphoma, Weihui, Henan, China

**Keywords:** myeloproliferative neoplasms, thrombotic events, clinical characteristics, risk factors, retrospective analysis

## Abstract

Thrombotic events are one of the main factors affecting the survival of patients with Philadelphia chromosome-negative (Ph-) myeloproliferative neoplasms (MPNs). Therefore, a comprehensive understanding of the early relevant high-risk factors makes sense for early prevention and reducing mortality in these patients. In this study, we conducted a retrospective analysis of 336 patients with Ph- MPN and summarized the clinical characteristics, incidence of thrombotic events and influencing factors. Thrombotic events occurred in 27.7% (93/336) of patients. Among the thrombotic events, arterial thrombosis occurred in 86 cases (92.5%), the most common thrombotic event was cerebral infarction (69/93, 74.2%). Univariate analysis and logistic regression identified that diagnosis of Polycythemia Vera (PV)/Essential Thrombocythemia (ET), thrombotic events before diagnosis and D-dimer≥1mg/L were the independent risk factors for thrombotic events at initial diagnosis in MPN patients (*P*<0.05). Receiver operating characteristic (ROC) curve analysis revealed that the integrated predictive efficacy of the triple-variable combination was markedly superior to that of any single parameter alone, yielding a sensitivity of 72.04% (95% CI: 61.8%-80.9%), a specificity of 74.49% (95% CI: 68.5%-79.8%), and an area under the curve (AUC) of 0.771 (95% CI: 0.723 - 0.815). Additionally, univariate analysis further identified smoking history, elevated hemoglobin (Hb≥136g/L), hematocrit (HCT≥0.42), D-dimer-to-fibrinogen ratio (DFR≥0.243) and JAK2^V617F^ mutation as potential risk factors for thrombosis (*P*<0.05), necessitating validation in future studies. These findings facilitate the early identification of Ph-MPN patients at heightened risk for thrombotic events, enabling the implementation of targeted prophylactic strategies to mitigate thrombotic risk.

## 1 Introduction

Myeloproliferative neoplasms (MPNs) are malignant disorders of the hematopoietic system, which are characterized by the clonal proliferation of one or more myeloid cell lineages, resulting in a group of myeloid neoplasms. Clinically, MPN are manifested by hyperplasia of one or more types of blood cells, and are often accompanied by hepatomegaly, splenomegaly, or lymphadenopathy. Polycythemia vera (PV), essential thrombocythemia (ET), and primary myelofibrosis (PMF) are collectively referred to as Philadelphia chromosome-negative (Ph-) MPN (1).

Thrombotic events, hemorrhagic events and leukemia transformation are the main factors influencing the survival of MPN patients. Approximately 35%-70% of MPN patients die due to thrombotic events, which seriously affect the prognosis and quality of patients’ life (2, 3). Hence, it is of extreme significance to be capable of identifying high-risk groups of thrombotic events at an early stage and intervene promptly. Studies have shown that factors such as age, smoking history, peripheral blood cell count, history of thrombosis, cardiovascular risk factors and driver genes are all regarded as potential high-risk factors for thrombotic events in MPN. However, the high-risk factors vary across different studies (4). Therefore, further large-scale studies are still needed for clarification. In this study, the clinical data of 336 Ph-MPN patients initially diagnosed in our hospital were retrospectively analyzed to explore the correlation between clinical characteristics and the occurrence of thrombotic events, so as to provide a basis for early clinical prevention and treatment of thrombosis.

## 2 Methods

### 2.1 Patients

**Figure 1:**
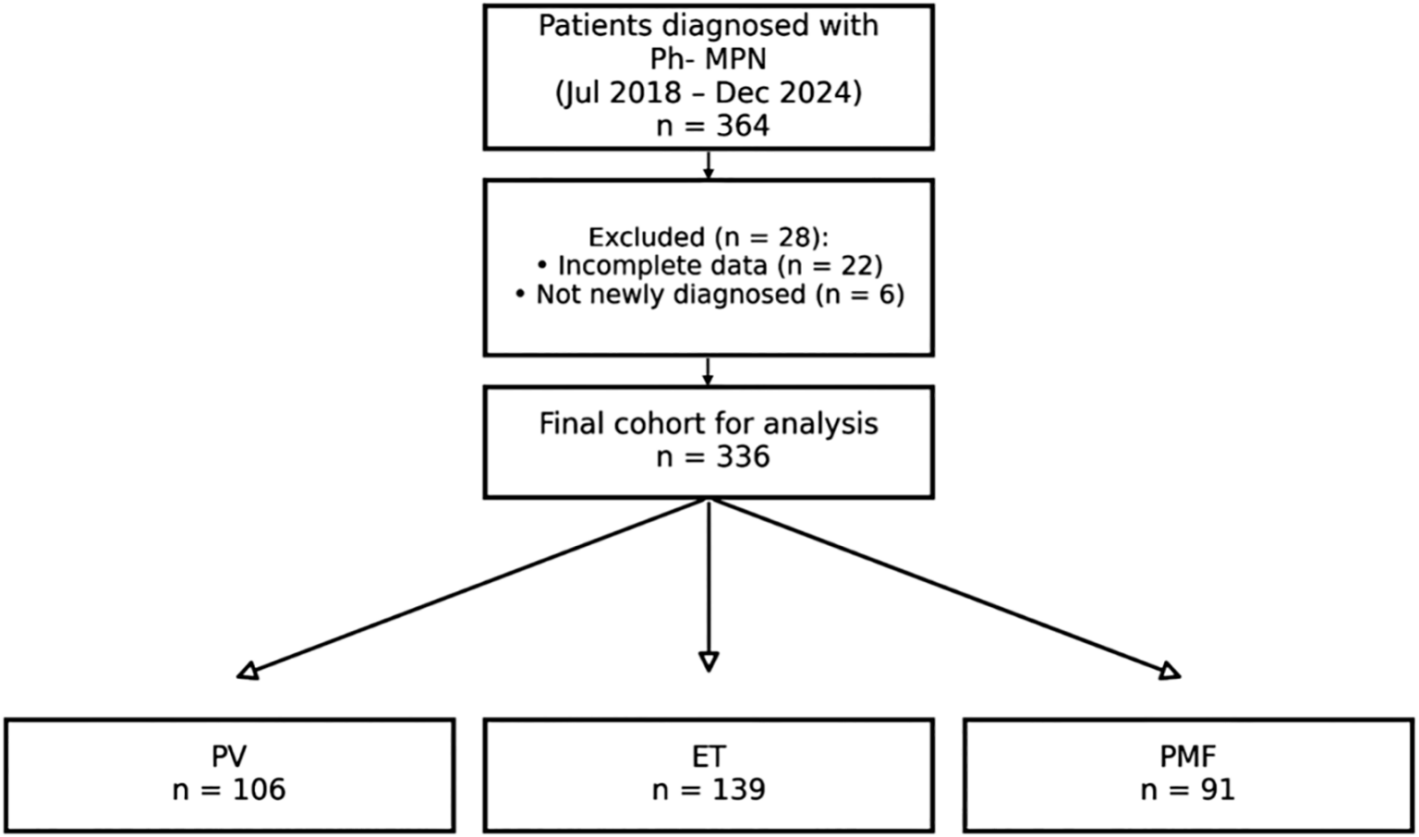
Flow diagram of patient selection and classification. A total of 364 patients diagnosed with Philadelphia chromosome-negative myeloproliferative neoplasms (MPN) at the First Affiliated Hospital of Xinxiang Medical University between July 2018 and December 2024 were screened. After exclusion of 28 patients (22 with incomplete clinical data and 6 who were not newly diagnosed), 336 patients constituted the final study cohort. These were classified into three diagnostic subtypes: polycythemia vera (PV, n = 106), essential thrombocythemia (ET, n = 139) and primary myelofibrosis (PMF, n = 91). MPN, myeloproliferative neoplasm; PV, polycythemia vera; ET, essential thrombocythemia; PMF, primary myelofibrosis.

A total of 336 patients with newly diagnosed and complete clinical data of Ph-MPN who were hospitalized in the Department of Hematology of the First Affiliated Hospital of Xinxiang Medical University from July 2018 to December 2024 were included. All patients met the diagnostic criteria of Ph-MPN in the 2016 version of WHO (5). All patients received risk-adapted therapy (such as phlebotomy and/or hydroxyurea) according to the NCCN Clinical Practice Guidelines in Oncology for Myeloproliferative Neoplasms during their treatment period (2018 – 2024). PV patients maintained hematocrit (HCT) <0.45 via phlebotomy and/or hydroxyurea. Low-risk patients received aspirin (100 mg/day) or phlebotomy when indicated. High-risk patients received hydroxyurea or interferon alfa-2a/2b. HCT was measured monthly during induction and quarterly during maintenance, with adjustments based on stability. This study was approved by the Ethics Committee of the First Affiliated Hospital of Xinxiang Medical University (Ethics approval number: EC - 025-332). A total of 364 consecutive patients with newly diagnosed Ph-negative MPN admitted between July 2018 and December 2024 were screened. After exclusion of 28 patients with incomplete data or non-incident disease, 336 patients met all eligibility criteria and were included in the final analysis (**Figure 1**).

### 2.2 Clinical data collection

The clinical data including the patients’ gender, age, onset symptoms, medical history, smoking history, driver gene mutation status, blood routine, coagulation function, blood lipid, myocardial enzyme spectrum, spleen enlargement status, the occurrence of hemorrhagic events and thrombotic events of all patients with Ph-MPN were collected. Spleen enlargement was precisely diagnosed through examinations such as abdominal ultrasound, CT and MRI. Thrombotic and hemorrhagic events were defined as those occurring within 3 months before or after the initial diagnosis. Among prior events (*n* = 106), the median time to diagnosis was 0.5 months (range: 0.1 - 3.0 months). For post-diagnosis even *n* = 4), the median time from diagnosis to event occurrence was 0.2 months (range: 0.1 - 0.4 months). Thrombotic events were defined as arterial or venous thrombosis accurately diagnosed by examinations such as vascular ultrasound, CT, CTA and MRI. Arterial thrombosis encompassed cerebral infarction, myocardial infarction, splenic infarction, lower extremity arterial occlusion and venous thrombosis included abdominal portal vein thrombosis, lower extremity venous thrombosis, etc. Hemorrhagic events were skin and mucous membrane bleeding with clinical manifestations (such as skin ecchymosis, gum bleeding, nasal bleeding), gastrointestinal bleeding, cerebral hemorrhage confirmed by imaging examinations, etc.

### 2.3 Statistical analysis

This study employed a retrospective analysis approach to statistically analyze the clinical characteristics, the occurrence of thrombotic events and hemorrhagic events in patients with Ph-MPN, and to analyze the correlation between the clinical characteristics and the occurrence of hemorrhagic events and thrombotic events. For measurement data, normal distribution test and homogeneity of variance test were conducted. For measurement data with non-normal distribution, median (range) and Mann-Whitney U non-parametric test were utilized; for categorical variable data, chi-square test or Fisher’s exact test was employed for testing; the regression analysis employed binary logistic regression with backward stepwise selection, variables from **Table 1** with univariate *P* < 0.05 were entered, and removal was based on likelihood ratio test *P* ≥ 0.10. Receiver operating characteristic (ROC) curve analysis was performed to determine the area under the curve (AUC). The analysis was performed through SPSS25.0 software, *P*< 0.05 was regarded as the criterion for statistically significant differences.

**Table 1:** Analyze of the risk factors of thrombotic events in patients with MPN.

Risk Factors	Thrombotic events (n=93)	No thrombotic events (n=243)	*P*
Male, n (%)	50 (53.8%)	100 (41.2%)	0.037*
Age median (range)	65 (32 - 82)	60 (18 - 91)	0.027*
Age≥60, n (%)	62 (66.7%)	125 (51.4%)	0.012*
WBC,10^9^/L median (range)	10.0 (1.0 - 48.9)	10.0 (1.2 - 90.9)	0.998
RBC,10^12^/L median (range)	5.3 (1.8 - 9.8)	4.5 (1.1 - 10.5)	0.003**
RBC≥4.7×10^12^/L, n (%)	58 (62.4%)	110 (45.3%)	0.005**
Hb, g/L median (range)	152 (55 - 220)	131 (41 - 232)	0.002**
Hb≥136g/L, n (%)	61 (65.6%)	107 (44.0%)	<0.001***
HCT,% median (range)	47.5 (18.5 - 74.6)	40.6 (12.8 - 73.9)	0.001**
HCT≥42%, n (%)	59 (63.4%)	109 (44.9%)	0.002**
PLT,10^9^/L median (range)	669 (24 - 1701)	689 (3 - 2498)	0.970
NLR median (range)	5.02 (0.67 - 99.64)	5.0 (0.63 - 43.45)	0.834
LDH, U/L median (range)	270 (139 - 4690)	288 (127 - 1820)	0.129
LDH≥245U/L, n (%)	52 (55.9%)	154 (63.4%)	0.209
D-dimer, ug/ml median (range)	0.7 (0.1 - 8.7)	0.6 (0.0 - 7.6)	<0.001***
D-dimer≥1mg/L, n (%)	32 (34.4%)	35 (14.4%)	<0.001***
FIB, g/L median (range)	2.53 (1.07 - 5.24)	2.54 (0.98 - 7.27)	0.866
DFR	0.284 (0.049 - 2.695)	0.230 (0.008 - 2,937)	<0.001***
DFR≥0.243, n (%)	59 (63.4%)	109 (44.9%)	0.002
Hyperlipidemia, n (%)	14 (15.1%)	48 (19.8%)	0.320
Splenomegaly, n (%)	53 (57.0%)	149 (61.3%)	0.469
Smoking, n (%)	31 (33.3%)	45 (18.5%)	0.004**
Diabetes, n (%)	9 (9.7%)	24 (9.9%)	0.956
Hypertension, n (%)	51 (54.8%)	113 (46.5%)	0.171
Coronary Heart Disease, n (%)	16 (17.2%)	22 (9.1%)	0.035*
Myocardial infarction, n (%)	9 (9.7%)	4 (1.6%)	0.002**
Cerebral infarction, n (%)	51 (54.8%)	46 (18.9%)	<0.001***
Thrombotic events before diagnosis, n (%)	58 (62.4%)	50 (20.6%)	<0.001***
Diagnosis			0.005**
PV, n (%)	38 (35.8%)	68 (64.2%)	
ET, n (%)	41 (29.5%)	98 (70.5%)	
PMF, n (%)	14 (15.4%)	77 (84.6%)	
JAK2^V617F^ mutation, n (%)	74 (79.6%)	164 (67.5%)	0.029*
CALR mutation, n (%)	6 (6.5%)	32 (13.2%)	0.082
MPL mutation, n (%)	4 (4.3%)	6 (2.5%)	0.599

Data are shown as median (range) or number (percentage). Group differences were assessed with the Mann–Whitney U test for continuous variables and the χ²/Fisher exact test for categorical variables. Abbreviations are identical to those listed in **Table 2**. *P < 0.05, **P < 0.01, ***P < 0.001 (thrombotic *vs*. non-thrombotic group).

## 3 Results

### 3.1 Clinical features of patients with Ph-MPN

This study included a cohort of 336 patients with Ph-MPN, comprising 150 male and 186 female participants. The median age was 63 (18~91) years old, and patients aged ≥ 60 years accounted for 55.7%. According to the classification by subtypes, PV, ET and PMF accounted for 31.5% (106/336), 41.4% (139/336) and 27.1% (91/336) respectively. There were no statistically significant differences in age and gender distribution among the three subtypes. Significant differences were observed in blood cell levels: the white blood cell (WBC) count in PV patients was significantly higher than that in the ET patients (12.1×10^9^/L *vs* 9.6×10^9^/L, *P* = 0.004). Although the WBC count in PV patients was also higher than that in the PMF patients, the difference did not reach statistical significance (12.1×10^9^/L *vs* 9.5×10^9^/L, *P* = 0.052). No significant difference was found in WBC count between ET and PMF patients (*P >* 0.05). Regarding red blood cell parameters, PV patients had significantly higher red blood cell (RBC) counts, hemoglobin (Hb) levels and hematocrit (HCT) compared to ET and PMF patients (*P* < 0.05). In terms of platelet count, ET patients showed significantly higher levels than PV (904×10^9^/L *vs* 472×10^9^/L, *P <* 0.001) and PMF patients (904×10^9^/L *vs* 459×10^9^/L, *P <* 0.001). Additionally, the neutrophil-to-lymphocyte ratio (NLR) was significantly higher in PV and PMF patients compared to ET patients (5.9 *vs* 4.32, *P* {it} = {/it}0.004; 6.76 *vs* 4.32, {it}P = {/it}0.002) (**Table 2**).

**Table 2:** Clinical characteristics of 336 patients with MPN.

Characteristics	Total (n=336)	PV (n=106)	ET (n=139)	PMF (n=91)	*P*
Gender, n (%)					0.159
Male	150 (44.6%)	54 (50.9%)	54 (38.8%)	42 (46.2%)	
Female	186 (55.4%)	52 (49.1%)	85 (61.2%)	49 (53.8%)	
Age median (range)	63 (18 - 91)	62 (18 - 79)	64 (20 - 91)	62 (38 - 84)	0.539
<60, n (%)	149 (44.3%)	45 (42.5%)	63 (45.3%)	41 (45.1%)	0.893
≥60, n (%)	187 (55.7%)	61 (57.5%)	76 (54.7%)	50 (54.9%)	
WBC, 10^9^/L median (range)	10.0 (1.0 - 90.9)	12.1 (1.0 - 39.8)	9.6 (1.2 - 90.9)	9.5 (1.8 - 59.1)	0.016*
RBC,10^12^/L median (range)	4.7 (1.1 - 10.5)	6.9 (2.4 - 10.5)	4.4 (1.1 - 7.8)	3.8 (1.4 - 9.3)	<0.001***
Hb, g/L median (range)	136 (41 - 232)	185 (102 - 232)	130 (48 - 205)	109 (41 - 177)	<0.001***
HCT,% median (range)	42.0 (12.8 - 74.6)	58.2 (30.5 - 74.6)	40.0 (14.7 - 48.1)	34.2 (12.8 - 69.4)	<0.001***
PLT,10^9^/L median (range)	684 (3 - 2498)	472 (10 - 2206)	904 (93 - 2498)	459 (3 - 2372)	<0.001***
NLR median (rang)	5.01 (0.63 - 99.64)	5.90 (0.67 - 21.78)	4.32 (0.94 - 99.64)	6.76 (0.63 - 43.45)	0.002**
LDH, U/L median (range)	280 (127 - 4690)	274 (128 - 770)	251 (143 - 733)	408 (127 - 4690)	<0.001***
D-dimer, ug/ml median (range)	0.6 (0.0 - 8.7)	0.5 (0.0 - 8.7)	0.6 (0.1 - 6.5)	0.7 (0.2 - 4.2)	0.004**
FIB, g/L median (range)	2.54 (0.98 - 7.27)	2.23 (1.02 - 4.82)	2.61 (1.16 - 7.27)	2.80 (0.98 - 6.11)	<0.001***
DFR	0.243 (0.008 - 2.937)	0.240 (0.008 - 2.937)	0.229 (0.029 - 2.695)	0.271 (0.060 - 1.464)	0.082
Hyperlipidemia, n (%)	62 (18.5%)	19 (17.9%)	25 (18.0%)	18 (19.8%)	0.929
Splenomegaly, n (%)	202 (60.1%)	79 (74.5%)	56 (40.3%)	67 (73.6%)	<0.001***
Smoking, n (%)	76 (22.6%)	21 (19.8%)	33 (23.7%)	22 (24.2%)	0.703
Diabetes, n (%)	33 (9.8%)	13 (12.3%)	10 (7.2%)	10 (11.0%)	0.380
Hypertension, n (%)	164 (48.8%)	74 (69.8%)	47 (33.8%)	43 (47.3%)	<0.001***
Coronary Heart Disease, n (%)	38 (11.3%)	14 (13.2%)	15 (10.8%)	9 (9.9%)	0.741
Myocardial infarction, n (%)	13 (3.9%)	3 (2.8%)	8 (5.8%)	2 (2.2%)	0.313
Cerebral infarction, n (%)	97 (28.9%)	40 (37.7%)	37 (26.6%)	20 (22.0%)	0.039*
Thrombotic events before diagnosis, n (%)	108 (32.1%)	42 (39.6%)	44 (31.7%)	22 (24.2%)	0.068
Thrombotic events at diagnosis, n (%)	93 (27.7%)	38 (35.8%)	41 (29.5%)	14 (15.4%)	0.005**
Types of Thrombus					0.112
Arterial, n (%)	86 (92.5%)	35 (92.1%)	39 (95.1%)	12 (85.7%)	
Venous, n (%)	4 (4.3%)	2 (5.3%)	2 (4.9%)	0 (0.0%)	
Arterial and Venous, n (%)	3 (3.2%)	1 (2.6%)	0 (0.0%)	2 (14.3%)	
Hemorrhagic events at diagnosis, n (%)	18 (5.4%)	3 (2.8%)	9 (6.5%)	6 (6.6%)	0.377
JAK2^V617F^ mutation, n (%)	238 (70.8%)	93 (87.7%)	86 (61.9%)	59 (64.8%)	<0.001***
CALR mutation, n (%)	38 (11.3%)	0 (0.0%)	24 (17.3%)	14 (15.4%)	<0.001***
MPL mutation, n (%)	10 (3.0%)	0 (0.0%)	6 (4.3%)	4 (4.4%)	0.093

Values are expressed as median (range) for continuous variables and number (percentage) for categorical variables. Inter-group comparisons were performed with the Kruskal–Wallis test (continuous data) or the χ²/Fisher exact test (categorical data). MPN, myeloproliferative neoplasm; PV, polycythaemia vera; ET, essential thrombocythaemia; PMF, primary myelofibrosis; WBC, white-blood-cell count; RBC, red-blood-cell count; Hb, haemoglobin; HCT, haematocrit; PLT, platelet count; NLR, neutrophil-to-lymphocyte ratio; LDH, lactate dehydrogenase; FIB, fibrinogen; DFR, D-dimer-to-fibrinogen ratio. *P < 0.05, **P < 0.01, ***P < 0.001 (overall comparison among the three diagnostic subtypes).

Analysis of coagulation parameters revealed significant differences in D-dimer and fibrinogen (FIB) levels across the three subtypes, while the D-dimer/fibrinogen ratio (DFR) did not demonstrate statistical significance. Notably, patients with PMF displayed elevated D-dimer concentrations compared to those with PV (0.7μg/mL *vs* 0.5μg/mL; *P* = 0.003) and ET (0.7μg/mL *vs* 0.6μg/mL; *P* = 0.004). No significant intergroup variation was observed between PV and ET patients (*P* > 0.05). Both the ET and PMF patients demonstrated significantly higher FIB levels than the PV group (2.61 g/L *vs* 2.23 g/L, *P* < 0.001; 2.80 g/L *vs* 2.23 g/L, *P* < 0.001), but no significant difference was found between the ET and PMF groups (*P* > 0.05).

In terms of medical history, significant differences were observed in the prevalence of hypertension among patients with PV, ET, and PMF (*P*<0.001), with rates of 69.8%, 33.8% and 47.3% respectively. Pairwise comparisons revealed that the hypertension prevalence in PV patients was significantly higher than that in both ET and PMF patients (*P*<0.05). Furthermore, PV patients demonstrated a significantly higher prevalence of prior cerebral infarction compared to PMF patients (*P* = 0.039). Splenomegaly was observed in 60.1% (202/336) of Ph-MPN patients, with PV and PMF patients being more prone to splenomegaly than ET patients (*P*<0.05). In terms of genetic mutations, the overall incidence of JAK2^V617F^ mutation in Ph-MPN patients was 70.8% (238/336), with mutation rates of 87.7% (93/106) in PV, 61.9% (86/139) in ET, and 64.8% (59/91) in PMF patients. The incidence of JAK2^V617F^ mutation in PV patients was significantly higher than that in ET and PMF patients (PV *vs* ET, *P* = 0.000; PV *vs* PMF, *P* < 0.001). Furthermore, the incidence of CALR and MPL gene mutations was 11.3% (38/336) and 3.0% (10/336), respectively. The incidence of CALR mutations in ET and PMF patients was significantly higher than that in PV patients (ET *vs* PV, *P* < 0.001; PMF *vs* PV, *P* < 0.0010) (**Table 2**).

### 3.2 Thrombotic events and hemorrhagic events

Among the 336 Ph-MPN patients, 93 (27.7%) experienced thrombotic events at the time of diagnosis. Based on the type of thrombosis, these events were categorized into arterial thrombosis, venous thrombosis, and mixed thrombosis. Arterial thrombosis was the most common, accounting for 92.5% (86/93), while venous thrombosis was less frequent, representing 4.3% (4/93), and mixed thrombosis accounted for 3.2% (3/93) (**Table 2**). In terms of the vascular distribution of thrombotic events, cerebral arterial thrombosis was the most prevalent, occurring in 74.2% (69/93) of cases, followed by multi-site thrombosis (9.7%, 9/93) and coronary arterial thrombosis (7.5%, 7/93). Splenic arterial thrombosis and lower extremity arterial thrombosis had the same incidence rate of 3.2% (3/93), whereas thrombosis in the abdominal venous system was the least common, accounting for only 2.2% (2/93).

Regarding hemorrhagic events, 18 patients (5.4%, 18/336) exhibited bleeding manifestations at diagnosis, primarily including skin ecchymosis, gingival bleeding, epistaxis, gastrointestinal bleeding, and intracranial hemorrhage. Among these, skin and mucosal bleeding were the most common, with skin ecchymosis accounting for 38.9% (7/18), gingival bleeding for 27.8% (5/18), and epistaxis for 16.7% (3/18). Bleeding in critical organs was less frequent, with gastrointestinal bleeding and intracranial hemorrhage representing 11.1% (2/18) and 5.6% (1/18) of cases, respectively. It should be specifically noted that one patient experienced concurrent cerebral infarction (counted toward thrombotic events) and gastrointestinal bleeding (counted toward hemorrhagic events), with both events separately incorporated into their corresponding statistical analyses.

### 3.3 Analysis of risk factors for thrombotic events in patients with Ph- MPN

By comparing the clinical data of MPN patients with and without thrombotic events, the results showed that the risk of thrombosis was significantly increased in male patients and those aged ≥ 60 years (*P* < 0.05). Regarding medical history, among the 93 patients with thrombotic events, 58 (62.4%) had thrombotic events before diagnosis, which was significantly higher than that of the non-thrombotic group (62.4% *vs* 20.6%, *P* < 0.001). Additionally, patients with a history of smoking, coronary heart disease, myocardial infarction, and cerebral infarction were more likely to experience thrombotic events (*P* < 0.05), whereas diabetes and hypertension were not associated with thrombosis. When analyzed by diagnostic subtype, the incidence of thrombotic events in PV, ET, and PMF patients was 35.8%, 29.5% and 15.4% respectively. A statistically significant difference in the incidence of thrombotic events was observed among the three groups (*P* = 0.005). Pairwise analysis showed that the incidence of thrombotic events in PV and ET patients was significantly higher than that in PMF patients (PV *vs* PMF, *P* = 0.001; ET *vs* PMF, *P* = 0.014). Therefore, PV and ET diagnosis was included as an independent factor in the subsequent multivariate analysis. Regarding driver genes, the incidence of thrombotic events in patients with JAK2^V617F^ mutant was significantly higher than that in patients with wild-type (79.6% *vs* 67.5%, *P* = 0.029).

In terms of blood routine parameters, higher levels of RBC count, Hb and HCT were significantly associated with the occurrence of thrombotic events (*P* < 0.05). When stratified by median values, the proportion of patients with values above the median in the thrombotic group was significantly higher than the non-thrombotic group (RBC ≥ 4.7×10^1^²/L: 62.4% *vs* 45.3%; Hb ≥ 136g/L: 65.6% *vs* 44.0%; HCT ≥ 0.42: 63.4% *vs* 44.9%, all *P* < 0.05), while WBC, NLR and PLT were not associated with thrombosis (*P* > 0.05), suggesting that MPN patients with erythrocytosis are more prone to thrombotic events. Regarding coagulation function, the levels of D-dimer (0.7mg/L *vs* 0.6mg/L, *P* < 0.001) and DFR (0.284 *vs* 0.230, *P* < 0.001) were significantly higher in the thrombotic group. Additionally, the proportion of patients with D-dimer ≥ 1mg/L and DFR ≥ 0.243 was also significantly higher in the thrombotic group (*P* < 0.05), indicating that elevated D-dimer and DFR are closely related to thrombotic events. There was no significant difference in FIB levels between the two groups. Moreover, lactate dehydrogenase, splenomegaly, and dyslipidemia were not significantly associated with thrombotic events (*P* > 0.05) (**Table 1**).

Multivariate analysis incorporating variables including gender, age, diagnosis (PV+ET), coronary heart disease, thrombotic events before diagnosis, smoking history, Hb ≥ 136 g/L, HCT ≥ 0.42, D-dimer ≥ 1 mg/L, DFR ≥ 0.243, and JAK2^V617F^ mutation, revealed that PV/ET diagnosis (OR = 3.311, *P* = 0.004), thrombotic events before diagnosis (OR = 5.161, *P* < 0.001), and D-dimer ≥ 1 mg/L (OR = 3.360, *P* = 0.002) were independent risk factors for thrombotic events at initial diagnosis in MPN patients (**Table 3**).

**Table 3:** Multivariate regression analysis of risk factors for thrombotic events.

Predictors	OR	95%CI	*P*
Male	0.784	0.386~1.592	0.500
Age≥60	1.064	0.571~1.983	0.845
Diagnosis (PV + ET)	3.311	1.462~7.498	0.004**
Coronary Heart Disease	1.914	0.798~4.592	0.146
Thrombotic events before diagnosis	5.161	2.880~9.250	<0.001***
Smoking	1.736	0.785~3.837	0.173
Hb≥136g/L	1.925	0.444~8.343	0.381
HCT≥0.42	0.625	0.150~2.603	0.519
D-dimer≥1mg/L	3.360	1.534~7.360	0.002**
DFR≥0.243	1.241	0.645-2.386	0.518
JAK2^V617F^ mutation	1.574	0.771~3.212	0.213

Multivariate binary logistic regression was performed with backward stepwise selection; variables with P < 0.05 in univariate analysis were entered into the model. OR, odds ratio; CI, confidence interval. **P < 0.01, ***P < 0.001.

### 3.4 The predictive value of diagnosis (PV + ET), thrombotic events before diagnosis and D-dimer≥1 mg/L for thrombotic events in MPN patients

By assessing the predictive value of diagnosis (PV + ET), thrombotic events before diagnosis, and D-dimer≥1mg/L for thrombotic events, the study revealed distinct performance characteristics among the variables in terms of sensitivity and specificity. Diagnosis (PV + ET) exhibited a high sensitivity of 84.95% (95% CI: 76.0%-91.5%); however, its specificity was notably low at 31.69% (95% CI: 25.9%-37.9%), with an AUC of 0.583 (95% CI: 0.528 - 0.636), indicating limited predictive utility when used in isolation. D-dimer ≥ 1mg/L demonstrated a low sensitivity of 34.41% (95% CI: 24.9%-45.0%), but a high specificity of 85.60% (95% CI: 80.5%-89.8%), with an AUC of 0.600 (95% CI: 0.545 - 0.653), suggesting moderate predictive performance as a standalone marker. Thrombotic events before diagnosis displayed a sensitivity of 62.37% (95% CI: 51.7%-72.2%) and a specificity of 79.42% (95% CI: 73.8%-84.3%), with an AUC of 0.709 (95% CI: 0.657 - 0.757), reflecting robust predictive efficacy.

**Figure 2:**
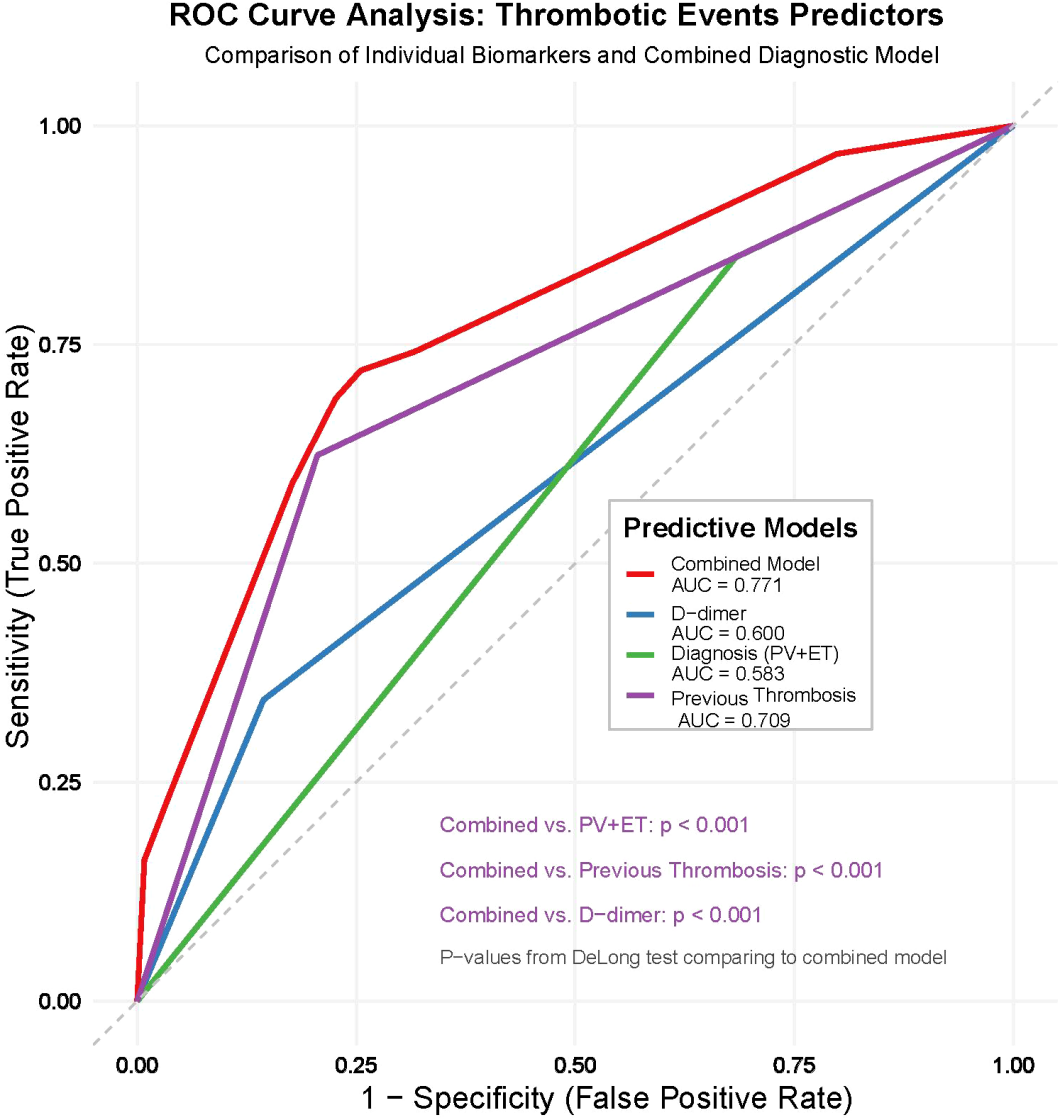
Receiver-operating-characteristic (ROC) curves for thrombotic-risk prediction in Philadelphia-negative MPN. The plot compares the predictive performance of three single variables—diagnosis subtype (PV + ET) (red), history of thrombotic events before diagnosis (green), and baseline D-dimer ≥ 1 mg L^−^¹ (blue)—with that of their combined model (black). Areas under the curve (AUCs) are 0.58 for diagnosis, 0.71 for prior thrombosis, 0.60 for D-dimer, and 0.77 for the three-variable combination. The grey diagonal indicates a non-informative test (AUC = 0.50). DeLong’s test showed that the combined model outperforms each individual predictor (all P < 0.001).

The combination of these variables significantly enhanced the predictive value, yielding a sensitivity of 72.04% (95% CI: 61.8%-80.9%), a specificity of 74.49% (95% CI: 68.5%-79.8%), and an AUC of 0.771 (95% CI: 0.723 - 0.815). This combined model outperformed any single variable, as evidenced by statistical significance (Z = 9.658, *P* < 0.001). These findings underscore that the integration of multiple variables markedly improves the predictive accuracy for thrombotic events, highlighting its substantial clinical utility (**Table 4**, **Figure 2**).

**Table 4:** The predictive value of diagnosis (PV + ET), thrombotic events before diagnosis and D-dimer≥1mg/L for thrombotic events in MPN patients.

Variable	Sensitivity	Specificity	AUC	*Z*	*P*
Diagnosis (PV + ET)	84.95 (76.0 - 91.5)	31.69 (25.9 - 37.9)	0.583 (0.528 - 0.636)	3.480	<0.001
Thrombotic events before diagnosis	62.37 (51.7 - 72.2)	79.42 (73.8 - 84.3)	0.709 (0.663 - 0.751)	7.357	<0.001
D-dimer≥1mg/L	34.41 (24.9 - 45.0)	85.60 (80.5 - 89.8)	0.600 (0.545 - 0.653)	3.675	<0.001
combination	72.04 (61.8 - 80.9)	74.49 (68.5 - 79.8)	0.771 (0.723 - 0.815)	9.658	<0.001

AUC, area under the receiver-operating-characteristic (ROC) curve; CI, confidence interval. Z values were calculated with DeLong’s test to compare AUCs. The “Combined” model includes diagnosis subtype (PV/ET), history of prior thrombosis, and D-dimer ≥ 1 mg L^−^¹. All *P* values for AUC comparisons were < 0.001.

## 4 Discussion

Thrombotic events represent one of the most prevalent complications in MPN. These events not only significantly impair patients’ quality of life but also alter the disease’s natural history by triggering progression to blast phase or overt myelofibrosis, which ultimately leads to shortened overall survival (6, 7). A meta-analysis of 13,436 newly diagnosed MPN patients revealed that the incidence of thrombotic events was approximately 20.0%, with rates of 28.6% for PV, 20.7% for ET, and 9.5% for PMF, respectively (8). Our study found that the overall incidence of thrombotic events in 336 newly diagnosed MPN patients was 27.7%. Among them, the incidence in PV, ET, and PMF patients showed significant differences, with rates of 35.8%, 29.5%, and 15.4%, respectively. Due to the higher levels of RBC and PLT counts, PV and ET patients were more prone to thrombosis, consistent with previous literature reports. A retrospective analysis conducted by a research team from the Institute of Hematology, Chinese Academy of Medical Sciences and the Second Affiliated Hospital of Tianjin Medical University, involving 1,537 MPN patients with JAK2^V617F^ mutation, reported that the incidence of thrombotic events was 43.9% (9). This higher incidence is likely attributable to the exclusive inclusion of patients with JAK2^V617F^ mutation. Furthermore, the study highlighted that arterial thrombosis was predominant, accounting for 91.4% of all cases, while venous thrombosis constituted 16.6%. In our study, arterial thrombosis accounted for 92.5% and venous thrombosis for 4.3%, which aligns closely with the findings reported in the literature.

Although studies suggest that gender, age, smoking history, hypertension, and thrombotic events before diagnosis may all serve as potential risk factors for thrombotic events in MPN patients, age ≥60 years and prior thrombotic events have been unequivocally established as independent risk factors (1, 10). Barbui further incorporated cardiovascular risk factors and the JAK2^V617F^ mutation into the IPSET thrombosis scoring system for predicting thrombotic risk in ET patients (11), while the other factors remain controversial. In this study, we analyzed associations between clinical characteristics (including gender, age, medical history) and thrombotic events in MPN patients. Univariate analysis revealed significant correlations between thrombotic events and male gender, age ≥60 years, smoking history, coronary artery disease, myocardial infarction, and thrombotic events before diagnosis. However, multivariate analysis identified only thrombotic events before diagnosis, PV/ET diagnosis, and elevated D-dimer as independent risk factors, while age, smoking history, coronary artery disease, and myocardial infarction showed no statistical significance. The observed statistical divergence likely stems from the study’s limited statistical power. Consequently, definitive confirmation of the putative associations between male gender, age, smoking, coronary artery disease, myocardial infarction, and thrombotic events in MPN necessitates rigorously designed prospective studies with adequate sample sizes, preferably employing multicenter cohort methodologies.

The JAK2^V617F^ mutation arises from the substitution of valine with phenylalanine at codon 617 within exon 14 of the JAK2 gene. This genetic alteration results in the aberrant activation of JAK2, subsequently leading to the activation of the JAK-STAT signaling pathway. The dysregulation of this pathway is the central mechanism underlying the pathogenesis of MPN. As documented in the literature, the prevalence of the JAK2^V617F^ mutation ranges from 70 - 90% in PV patients and 50 - 60% in ET and PMF patients (12). In our study, the incidence of the JAK2^V617F^ mutation was 87.7%, 61.9%, and 64.8% in PV, ET, and PMF patients, respectively, aligning closely with the previously reported data. The JAK2^V617F^ mutation status has been unequivocally established as an independent risk factor for thrombotic events in MPN patients (13, 14). Notably, emerging evidence suggests that a mutation allele burden ≥50% correlates with significantly elevated thrombotic risk (9), intensifying the focus on the clinical significance of the JAK2^V617F^ mutation burden. Our clinical observations indicate that MPN patients with JAK2^V617F^ mutation are more likely to experience thrombotic events. However, due to the limitations of detection methods, routine quantitative assessment of the JAK2^V617F^ mutation burden has not been implemented in MPN patients in our study. From a mechanistic standpoint, the JAK2^V617F^ mutation may facilitate thrombosis via several key pathways (15): overexpression of genes associated with inflammation, adhesion, and thrombosis in endothelial cells; suppression of P-selectin expression; activation of β1 and β2 integrins; upregulation of heparanase expression.

Elevated blood cell counts are associated with an increased risk of thrombosis in patients with MPN. Polycythemia has been demonstrated to contribute to thrombosis through mechanisms such as increasing blood viscosity (16, 17) studies on treated PV patients have demonstrated that maintaining HCT <0.45 significantly reduces the incidence of severe thrombotic events compared to HCT ≥0.45 (18), hence current guidelines recommend maintaining HCT <0.45 as the treatment target for PV patients. Consistent with existing literature, our analysis identified erythrocytosis-related parameters, including HCT ≥0.42, RBC ≥4.7×10¹²/L, and Hb ≥136 g/L, as risk factors for thrombotic events in MPN patients. Thrombocytosis and platelet dysfunction are common in MPN, but the relationship between platelet count elevation and thrombosis remains controversial. Although some studies reported increased thrombotic risk and shorter time to thrombosis in ET patients with PLT >593×10^9^/L (19), most evidence suggests no correlation between platelet levels and thrombotic events. Previous studies demonstrated that platelet counts in ET patients were not correlated with thrombotic risk; they exhibited a paradoxical U-shaped relationship with bleeding risk, with PLT ≥450×10^9^/L associated with a 3.7-fold increase in hemorrhagic complications, evidence reveals that MPN patients with markedly elevated platelet counts (PLT >1000×10^9^/L) paradoxically face greater risks of hemorrhagic manifestations than thrombotic events, a phenomenon attributed to impaired platelet function and acquired von Willebrand syndrome secondary to extreme thrombocytosis (20). These findings underscore current guidelines advocating platelet normalization as the therapeutic goal in cytoreductive therapy. Our study showed no association between platelet counts and thrombosis, which might be attributed to limitations in sample size or heterogeneity across MPN subtypes. Future investigations with larger cohorts and subtype-specific analyses are warranted to clarify these relationships and refine risk stratification strategies.

The relationship between leukocytosis and thrombotic events in patients with myeloproliferative neoplasms (MPN) remains controversial. Although multiple studies suggest that leukocytosis may be a significant risk factor for thrombosis in MPN patients, a comprehensive meta-analysis demonstrated a 59% increased thrombosis risk in MPN patients with leukocytosis compared to controls (OR = 1.59), with subgroup analysis revealing this association to be particularly pronounced in ET patients compared to PV patients, especially for arterial thrombosis (21). These findings are corroborated by the prospective REVEAL cohort study including 2,510 PV patients, which demonstrated that baseline WBC ≥11×10^9^/L was significantly associated with the risk of first thrombotic events (HR = 2.35, 95% CI 1.59 - 3.46), a multicenter retrospective study of 520 PV patients showed no statistically significant correlation between leukocytosis and thrombotic risk (P = 0.416), though it revealed strong associations with accelerated disease progression. Patients with WBC ≥15×10^9^/L exhibited 5.51-fold (95% CI: 1.55 - 19.58, P = 0.008) increased risks of disease transformation (22). Our cohort analysis revealed no statistically significant association between leukocytosis and thrombotic risk, a finding likely attributable to heterogeneous diagnostic thresholds for leukocytosis across studies and insufficient statistical power from the limited sample size to conduct stratified subgroup analyses by MPN subtypes. Therefore, the role of WBC in thrombosis remains debated. Future well-designed large-scale multicenter studies incorporating dynamic monitoring of peripheral blood parameters and long-term follow-up of thrombotic events are required to clarify the impact of WBC on thrombosis in MPN.

The relationship between coagulation abnormalities and thrombotic risk has been extensively investigated, with accumulating evidence indicating the prevalent hypercoagulable state in patients with MPN (23). Nevertheless, the prognostic significance of coagulation biomarkers in predicting thrombotic events among MPN patients remains incompletely characterized. D-dimer, a specific degradation product of cross-linked fibrin, has emerged as a sensitive biomarker for thrombosis. Elevated D-dimer levels not only reflect enhanced fibrinolytic activity but more importantly signify an imbalance in the coagulation-fibrinolysis homeostasis, establishing its clinical utility in thrombosis prediction (24) As the fundamental substrate in thrombogenesis, FIB undergoes thrombin-mediated enzymatic cleavage to form fibrin monomers. These monomers subsequently polymerize through cross-linking to establish the structural framework of thrombi, a process that concurrently activates the fibrinolytic system as a compensatory mechanism. The D-dimer-to-fibrinogen ratio (DFR), a composite parameter derived from these two biomarkers, serves as a more comprehensive assessment of the coagulation-fibrinolysis equilibrium. Emerging clinical evidence has validated DFR’s enhanced prognostic capacity over isolated parameter analysis in venous thromboembolism, particularly demonstrating significant correlations with the severity of thrombosis in patients with cerebral venous thrombosis (25, 26). Notably, the investigation of DFR in thrombosis in patients with MPN remains remarkably underexplored. Guo et al. (27) first documented significantly elevated DFR levels in MPN patients with thrombotic manifestations compared to non-thrombotic counterparts, suggesting its potential role in thrombotic risk assessment in MPN patients. However, this study did not concurrently assess the clinical implications of D-dimer elevation. Our investigation substantiates these observations. Univariate analysis revealed statistically significant increases in both D-dimer and DFR in the thrombotic cohort, and multivariate regression analysis further identified elevated D-dimer as an independent risk factor for thrombotic events in MPN patients.

It should be emphasized that in our study, D-dimer levels were assessed only once at the time of initial diagnosis and before the initiation of any treatment. This single baseline measurement was intended to capture the intrinsic hypercoagulable state of MPN patients, rather than reflect transient fluctuations due to disease progression or therapeutic interventions. While serial monitoring of D-dimer is subject to considerable variability and limited clinical interpretability, our results suggest that an elevated D-dimer at diagnosis can serve as a practical biomarker for identifying patients at higher risk of thrombosis. Thus, in our analysis, D-dimer was used solely for risk stratification at baseline, not as a marker for longitudinal follow-up. Further prospective studies with longitudinal sampling will be necessary to validate the clinical utility of this approach.

The IPSET-thrombosis model, which is widely recommended for risk stratification in ET by international guidelines, incorporates four variables: age >60 years, history of thrombosis, presence of JAK2^V617F^ mutation, and cardiovascular risk factors (28). In contrast, our current predictive model was based on diagnosis (PV/ET), history of prior thrombosis, and D-dimer level at diagnosis. Notably, while both models include history of thrombosis and age (directly in IPSET, indirectly in our multivariate analysis), our model did not retain JAK2^V617F^ mutation as an independent predictor after adjustment for diagnostic subtype. Instead, it introduced D-dimer as a novel laboratory biomarker reflecting baseline hypercoagulability. The inclusion of D-dimer, a routinely available and objective marker of coagulation activation, may offer additional value beyond clinical characteristics alone, especially in settings where comprehensive cardiovascular risk profiling is challenging or incomplete. However, unlike IPSET-thrombosis, our model does not incorporate cardiovascular risk factors or JAK2 mutation status as independent risk variables. Therefore, while our model demonstrated improved predictive accuracy (AUC = 0.771 for the combined model), direct comparison with IPSET or guideline-based tools requires prospective validation in larger and fully characterized cohorts.

Recent evidence from Duminuco et al. (29) further advances thrombotic risk assessment in MPN by evaluating the QRISK3 score, a validated cardiovascular risk calculator integrating factors such as age, blood pressure, diabetes, smoking, and lipid profile, in 935 ET and PV patients (29). Their analysis demonstrated that QRISK3 scores were significantly higher in high-risk groups (age >65 years or prior thrombosis), with a threshold of >7.5% providing good predictive performance for thrombotic events (sensitivity 65% and specificity 81% in ET; >5.5% in PV). Importantly, QRISK3 outperformed traditional models like IPSET-revised by better identifying hidden high-risk subgroups among low-risk patients and highlighting the benefits of cytoreductive therapy in high-QRISK3 groups. These findings complement our results, as both emphasize the value of multifaceted risk prediction: our model incorporates coagulation biomarkers (D-dimer) alongside diagnostic subtype and thrombosis history, while QRISK3 provides a more accurate integration of modifiable cardiovascular factors, which were not independently significant in our analysis but are prevalent in MPN (e.g., hypertension in 48.8% of our cohort). The alignment is particularly evident in PV and ET subgroups, where our thrombotic incidence (35.8% and 29.5%) mirrors Duminuco et al.’s focus, and elevated D-dimer in our study may correlate with the hypercoagulable state amplified by cardiovascular risks in QRISK3. However, differences in cohort composition (our Chinese population *vs*. their UK-based one) and endpoints suggest that adapting QRISK3 thresholds (e.g., to >7.0% based on our ROC data) could enhance its applicability in diverse settings. Integrating QRISK3 into our model could further refine risk stratification, potentially improving AUC beyond 0.771 by combining laboratory hypercoagulability markers with comprehensive cardiovascular profiling.

In future studies, integrating laboratory parameters such as D-dimer with established clinical risk factors, including those in QRISK3, may help to further refine individualized thrombosis risk stratification for MPN patients. A major limitation of our study, which is inherent to most retrospective analyses in MPN, is that all clinical and laboratory variables were collected only at diagnosis. As a result, our findings reflect the baseline risk profile and do not account for dynamic changes or modifications in risk factors that may occur over time with disease progression or treatment. Future prospective studies with longitudinal data collection are needed to more accurately assess the impact of evolving risk factors on thrombotic risk in MPN patients. A further limitation of our analysis, common to most retrospective MPN studies, is that all clinical and laboratory variables were collected only once at the time of diagnosis. Consequently, the present model captures the baseline risk profile but cannot account for dynamic changes in risk factors, such as blood-count normalization, emergence of new comorbidities, or clonal evolution, during follow-up or after therapeutic interventions. The inability to represent time-dependent modifications may lead to under- or over-estimation of thrombotic risk as the disease evolves. Prospective studies incorporating longitudinal sampling and time-varying covariate analyses will therefore be essential to determine how evolving risk factors influence thrombosis in MPN patients. Additionally, our study did not incorporate advanced cardiovascular risk tools like QRISK3, which could have strengthened the assessment of multifactorial risks; this represents an opportunity for validation in future cohorts.

In summary, multivariate analysis identifies prior thrombotic events, PV/ET subtypes, and elevated D-dimer levels as independent risk factors for thrombotic events in MPN patients. Further investigations are warranted to validate the thrombogenic potential of smoking history, elevated levels of Hb, HCT and DFR, and JAK2^V617F^ mutation. It should be noted that, in our multivariate analysis, the predictive significance of JAK2^V617F^ mutation for thrombotic events was lost after including diagnostic subtypes (PV/ET *vs*. PMF) as covariates. This suggests that the association between JAK2^V617F^ mutation and thrombosis may be driven predominantly by PV patients, where both the mutation rate and thrombotic risk are higher. The relatively lower mutation rate and risk of thrombosis in ET and PMF may weaken the independent effect of JAK2^V617F^ mutation in these subtypes. Thus, future studies with larger, subtype-specific cohorts are needed to more accurately evaluate the thrombogenic risk of JAK2^V617F^ mutation, particularly in ET patients. Therefore, MPN patients should undergo thrombosis risk evaluation at diagnosis through comprehensive assessment of clinical history and laboratory parameters, enabling the development of tailored prophylactic strategies to mitigate thrombotic complications.

Our predictive model, based on diagnosis subtype (PV/ET), history of prior thrombotic events, and baseline D-dimer level, uses clinical and laboratory parameters that are readily available at the time of diagnosis. Applying this model in clinical practice can help identify MPN patients at increased risk of thrombosis, allowing clinicians to tailor management strategies accordingly. High-risk patients may benefit from more intensive prophylactic interventions, including aggressive cytoreductive therapy, antiplatelet or anticoagulant agents, stricter control of cardiovascular risk factors, and closer clinical monitoring for thrombotic complications. The model’s simplicity and practicality make it well-suited for integration into routine workflows, supporting individualized and risk-adapted patient care. To ensure the broader applicability and reliability of our model, external validation is essential. We plan to conduct prospective validation studies in independent cohorts from multiple medical centers. In these future studies, the same variables will be systematically collected at diagnosis, and the model’s performance for predicting thrombotic events will be assessed using metrics such as area under the ROC curve, calibration plots, and decision curve analysis. Subgroup analyses will be performed to confirm the robustness of the model across different MPN subtypes and demographic groups. Successful external validation will support the integration of this risk stratification approach into daily clinical practice and contribute to improved, personalized management of patients with MPN.

## Data Availability

The original contributions presented in the study are included in the article/Supplementary Material. Further inquiries can be directed to the corresponding author.
